# Exploring Perceptions and Experiences of Patients Undergoing Transcranial Magnetic Stimulation (TMS) for Depression and Adjustment Disorder in Romanian Private Practices

**DOI:** 10.3390/medicina61040560

**Published:** 2025-03-21

**Authors:** Dan-Alexandru Constantin, Ionut-Horia Cioriceanu, Daiana Anne-Marie Constantin, Andrada-Georgiana Nacu, Liliana Marcela Rogozea

**Affiliations:** 1 Department of Fundamental, Prophylactic and Clinical Sciences, Faculty of Medicine, Transilvania University of Brasov, 500019 Brasov, Romania; englober@icloud.com (D.-A.C.); dr.cioriceanu@gmail.com (I.-H.C.); r_liliana@yahoo.com (L.M.R.); 2 Clinical Hospital of Psychiatry and Neurology Brasov, 500123 Brasov, Romania; 3 Department of Obstetrics and Gynecology, “Carol Davila’’ University of Medicine and Pharmacy, 050474 Bucharest, Romania; daiana-anne-marie.dimcea@drd.umfcd.ro

**Keywords:** transcranial magnetic stimulation (TMS), depression, adjustment disorders, patient perceptions, private healthcare, non-pharmacological treatment, mental health accessibility

## Abstract

*Background and Objectives*: Mental health disorders, including major depressive disorder and adjustment disorder with mixed anxiety and depressed mood, present a significant global burden, with early onset and progression leading to substantial individual and social impacts. While pharmacotherapy remains the standard treatment, many patients experience inadequate symptom relief or intolerable side effects. In this context, transcranial magnetic stimulation (TMS) has emerged as a non-invasive, well-tolerated neuromodulation technique offering an alternative treatment option. Although its clinical efficacy is well-documented, limited research exists on patient perceptions, decision-making processes and barriers to TMS utilization in private healthcare settings, particularly in Romania. This study explores patients’ experiences with TMS, factors influencing their treatment choices and comparative views on its acceptability relative to pharmacological interventions. *Materials and Methods*: A qualitative research design was employed, using semi-structured interviews with 20 patients diagnosed with MDD or AD who had undergone TMS therapy as part of two pilot studies which were non-randomized in Romanian private practices. Data were collected via interviews and analyzed thematically to identify patterns in patient perceptions, decision-making factors and treatment experiences. *Results*: Participants reported predominantly positive perceptions of TMS, citing improvements in mood, anxiety reduction, and enhanced daily functioning. The most common motivations for seeking TMS included dissatisfaction with pharmacotherapy, recommendations from physicians or peers and information obtained via online sources. TMS was perceived as a safer and more tolerable alternative to medication, particularly due to its lack of systemic side effects. However, barriers such as high treatment costs, limited insurance coverage and logistical challenges in accessing TMS services were noted as significant deterrents. *Conclusions*: The study highlights the strong preference for TMS among patients who seek alternatives to pharmacotherapy, with key motivators including efficacy, tolerability and non-invasiveness. However, systemic barriers to access remain a critical challenge in private healthcare settings. Future research should focus on expanding accessibility, improving patient education and integrating TMS into broader mental healthcare frameworks to optimize treatment outcomes.

## 1. Introduction

Mental health disorders represent a global cause for disability, with an increasing prevalence during recent years, according to Global Burden of Disease Study (GBD) [1]. Not only do these disorders cause a major burden on the population, but their onset occurs in youth, at around the age of 14 years [2]. Given the early onset and frequent chronic nature of mental disorders, they can disrupt the transition to a healthy adulthood and affect long-term productivity. They are also associated with high rates of school dropout, reduced economic productivity, suicide and homelessness, among other consequences that impact individuals, families and society [3,4].

Depressive disorders constitute major public health issues around the world, affecting 1.95% of the population of all ages across the globe [5], with a significant impact on morbidity and disability. Romanian people also suffer from a variety of these conditions, but face various challenges such as discrimination, lack of resources, and the mental health disparity in accessing quality treatment. While psychotherapy and pharmacotherapy are commonly seen approaches, many patients feel resistant to treatment or have intolerable side effects [6]. Even if stress-related disorders are categorized as other mental health disorders by GBD, they cause significant distress among individuals across the globe [5]. Adjustment disorder (AD) is a very common psychiatric disease that stands between normal reactions of individuals to stress and major psychiatric disorders [7]. Because of the diagnosis limitations of AD, its prevalence in global population varies from 1% to 2% according to the diagnosis tools used [8,9]. A high rate of AD prevalence and its correlation with a young age of onset make it an important disease that needs to be focused on. Risk factors include people living in urban areas, low educational level, and lack of social support. The most common type of this disorder is the AD type with a mixed anxiety and depressed mood, which is caused by several stressors like domestic problems, love affairs and illness [10].

Several therapeutic interventions are used to ease the burden of depressive disorders and AD. The most common therapeutic interventions are psychotherapy and drug treatment. Even if there are a lot of therapeutic approaches available, a high percentage of patients do not tolerate, are treatment-resistant, have adverse events or refuse traditional treatment approaches. Considering traditional drug treatments’ limitations and the interests of developing alternative non-invasive approaches, neuromodulation techniques, including transcranial magnetic stimulation (TMS) and transcranial direct electric stimulation (tDCS), are new emerging therapies that can be taken into consideration for treating depression and AD with mixed anxiety and depressed mood [11].

TMS has emerged as a promising treatment modality for depression and other psychiatric disorders, offering a non-invasive alternative for individuals who may not respond to traditional therapies or medication. By delivering magnetic pulses to specific regions of the brain, TMS aims to modulate neural activity and alleviate symptoms associated with mood disorders [12]. The TMS technique can decrease or increase cortical excitability, depending on the stimulation parameters. Repetitive transcranial magnetic stimulation (rTMS) with low frequency and continuous theta burst stimulation (cTBS) with low frequency can produce a long-term depression of neurons, while rTMS with a high frequency of intermittent theta burst stimulation (iTBS) can potentiate long-term neuronal activity. TMS is known for its capacity to balance the cortical excitability, stimulate neuroplasticity and secrete specific neurotransmitters, and can modify the cerebral flow into the brain [13,14,15].

In recent years, the application of TMS in treating depression has garnered considerable attention in both clinical and research settings. Its efficacy, coupled with its relatively low side-effect profile, has positioned TMS as a viable option, particularly for patients who have not experienced relief from other interventions [16]. The United States Food and Drug Administration (FDA) has approved TMS for treatment-resistant depression in 2008 [17], iTBS in 2018 [18], depression with anxiety in 2022 [19] and in 2024, it extended the upper age limit from 68 to 86 years old [20]. Other FDA-approved protocols are for obsessive–compulsive disorder (OCD) [21] and smoking cessation [22]. In Europe, experts categorize the TMS recommendations by levels of efficacy. Level A of efficacy is attributed to depression, while posttraumatic stress disorder is attributed to level B of efficacy [23].

TMS poses as a promising non-invasive alternative treatment for depression and adjustment disorders, offering benefits for people who do not respond well to conventional treatments. There are few studies available regarding the patients’ point of view, the way it is perceived, and the decision-making procedure in Romanian private clinics. Existing research pays more attention to effectiveness and safety of TMS, as compared to the role of the patient context [24]. However, patients’ acceptance of and the factors supporting TMS utilization are hardly paid any attention in those studies. Therefore, investigations must be conducted to explore the daily lives of patients, and their perceptions of being subjected to TMS for depression and AD in private health facilities in Romania.

Numerous studies have investigated patient perceptions of TMS effectiveness and acceptability across different cultural and healthcare contexts worldwide. Research suggests that patients’ attitudes towards TMS vary depending on factors such as prior treatment experiences, cultural beliefs and socioeconomic status. In a study conducted by [25], patients undergoing TMS treatment for depression in Australia reported positive perceptions of the therapy’s effectiveness, particularly in terms of symptom reduction and overall improvement in mood. However, cultural factors play a significant role in shaping patient perceptions of mental health treatment, including TMS, in Romania. Cultural attitudes toward mental illness, stigma and beliefs about treatment efficacy can influence patients’ willingness to seek help and engage in treatment [26]. Understanding these cultural nuances is essential for tailoring mental health interventions to meet the needs of diverse patient populations.

Understanding patient perspectives on TMS is paramount, particularly in the context of Romanian private healthcare practices. In Romania, where mental health services can vary in availability and quality, exploring patient perceptions can shed light on the acceptability, effectiveness and accessibility of TMS as a treatment option [27]. This exploration becomes even more crucial given the stigma surrounding mental health issues in many societies, including Romania, which may influence treatment-seeking behaviors and decision-making processes. The private healthcare sector in Romania plays a significant role in providing mental health services, offering patients a range of treatment options beyond those available in the public sector [26]. Insights from mining patient perspectives within the private healthcare context can offer insights into the dynamics of mental health treatment utilization, preferences and barriers to access.

This study tries to fill the literature gap in this field; thereby, the objective of our qualitative research is to investigate patient perspectives on transcranial magnetic stimulation (TMS) as a treatment option for major depressive disorder (MDD) and AD with mixed anxiety and depression mood in Romanian private practices, focusing on their experiences, perceptions and decision-making processes. To achieve this objective, the study focuses on the following key research questions: (1) how do patients with major depressive and AD with mixed anxiety and depressed mood perceive the effectiveness and acceptability of transcranial magnetic stimulation (TMS) in the Romanian private healthcare context?; (2) what factors influence patients’ decision-making processes regarding the utilization of transcranial magnetic stimulation (TMS) for treating depression and AD with mixed anxiety and depressed mood in Romanian private practices? The findings obtained from this study guide practical approaches, improve patient–provider interaction and contribute to the development of such policy initiatives for TMS treatment services intended to promote mental health care in Romanian private practice. By identifying the key factors that shape patient attitudes and treatment choices, the study could enhance mental health delivery care in Romanian private practice, ultimately promoting the adoption and optimization of TMS as a viable therapeutic option.

## 2. Materials and Methods

### 2.1. Design

For this study, a semi-structured interview was conducted with a subset of patients who participated in two pilot-controlled, non-randomized studies that included patients from Romanian private practices who were diagnosed with MDD and AD with mixed anxiety and depressed mood and underwent rTMS procedures.

The first study aimed to investigate the efficacy of rTMS in adult patients with MDD from a Romanian private practice, and assess whether concurrent psychotropic medication use negatively affects the TMS therapy outcome. Patients included in this study had to be diagnosed with MDD according to DSM-5 criteria [28], sign the informed consent and not have contraindications for TMS after completing the safety questionnaire [24]. TMS protocol consisted of stimulation over the left dorsolateral prefrontal cortex at a frequency of 10Hz, 100% motor threshold individual intensity, 15 trains, 100 pulses per train with a total of 1500 pulses. Study participants had to complete 20 treatment sessions for 4 weeks [29]. The second pilot-controlled, non-randomized study explored the efficacy and safety of rTMS in the treatment of AD with mixed anxiety and depressed mood on a group of patients from a Romanian private practice. It included patients over 18 years of age who were diagnosed with the subtype AD mixed anxiety and depressed mood according to DSM-5 [28], signed the informed consent, did not present contraindications after completing TMS questionnaire [24] and were treated with 10 sessions of rTMS over a 2-week period. The protocol used for this study included the following parameters: frequency 1 Hz, 110% stimulation intensity from individual motor threshold, inter-train interval 1000 ms, 1800 pulses, total stimulation time 30 min delivered over the right dorsolateral prefrontal cortex [30]. Patients with other concomitant major psychiatric disorders, non-treated somatic comorbidities and suicidal ideation were excluded from both studies. Depression and anxiety symptoms severity were assessed using the Hamilton Depression Rating Scale (HDRS) and anxiety symptoms were assessed with the Hamilton Anxiety Rating Scale (HAM-A) before and after the rTMS sessions were performed for each study. Both quantitative studies and the qualitative study received the ethics committee’s approval no. 1.3/21 May 2018, issued by the local Ethics Committee for Medical Scientific Research of the Faculty of Medicine of Transilvania University.

In this qualitative study, 20 participants were included (*n* = 20) from both quantitative studies who completed all rTMS treatment sessions (10 sessions for AD, 20 sessions for MDD), signed the informed consent and were willing to participate in a semi-structured interview. Patients who refused to participate at the interview were excluded from the study.

### 2.2. Study Instrument

To gather the relevant information from the patients included in our qualitative study, we designed a semi-structured interview with 11 questions, which was created in a manner to help researchers collect information in alignment with the research objectives, research questions and title of the study.

**Data Collection**

Participants who completed the mandatory rTMS interventions from each study were asked to be interviewed. After their consent was given, the semi-structured interviews were undertaken on a single occasion, using Zoom or at the clinic according to patients’ choice. A male researcher (IHC) took the interviews. Patients were informed about the purpose of the interview and the interviewer’s background. Each interview was audio recorded, lasted between 8–20 min and was conducted in the Romanian language.

### 2.3. Data Analysis

**Figure 1:**
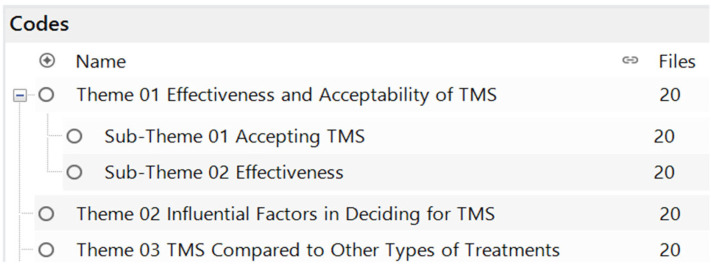
List of themes and sub-themes.

All interviews were transcribed and translated into English, and read by two researchers (DAMC and AGN) several times for the identification of similarities and models in participants’ answers. The qualitative data were analyzed thematically with the support of NVivo 14 software by QSR International, trial version to get a better understanding of the problems and have some sort of definitive answers to the pre-defined research questions. The open coding of data provided the following themes and sub-themes (Figure 1), which are provided below and analyzed individually with the support of annotation and transcripts from interviews.

## 3. Results

A total of 20 participants (n = 20) were included in the study and were subject to their views being gathered through the semi-structured interview protocol. The participants were given pseudonyms from P01 to P20 to keep their anonymity. Demographic information on the subjects along with their codes is provided below. By analyzing the sex ratio, we can observe that, from all 20 participants, 18 are female and only 2 are male. The codes and sex ratio are presented in Table 1.

From the total group, 10 patients were diagnosed with MDD and 10 patients were diagnosed with a subtype of AD with mixed anxiety and depressed mood, according to the quantitative study’s design. The mean age for the participants with MDD was 42.90, ranging from 21 to 64 years. Participants from the study with AD with a mixed anxiety and depressed mood had a mean age of 33 years, ranging from 25 to 44 years (Table 2).

**Theme 1: Effectiveness and Acceptability of TMS**

**Figure 2:**
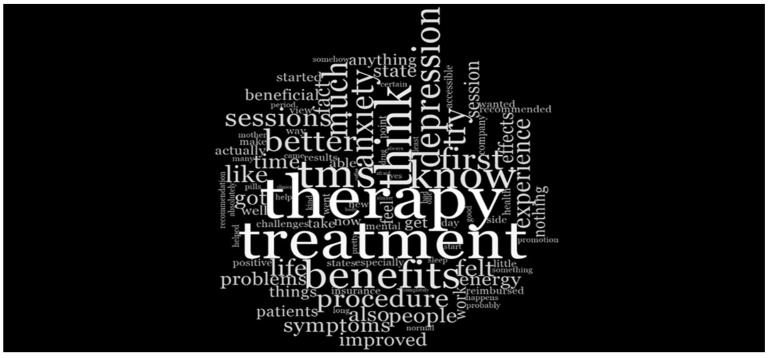
Word cloud for Theme 1, showing frequency of amalgamated coding.

The first theme of the study answered the research question “How do patients with major depressive and AD with mixed anxiety and depressed mood perceive the effectiveness and acceptability of transcranial magnetic stimulation (TMS) in the Romanian private healthcare context?” The responses provided insight into accepting the TMS as a treatment for depression and AD with mixed anxiety and depressed mood, and how this treatment has influenced their daily life. A word cloud generated for this theme is presented in Figure 2.

**Sub-Theme 1: Accepting TMS**

**Figure 3:**
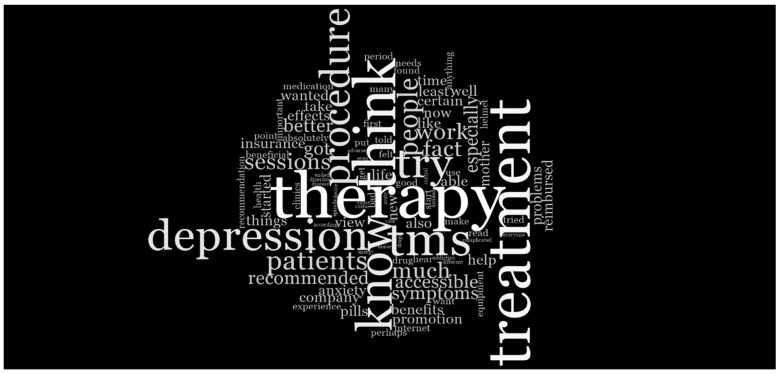
Word cloud for Sub-Theme 1, showing frequency of coded words.

It has been observed in the responses that almost all the participants felt bad because of their depression, feelings of sadness, loss of interest, fatigue, difficulty concentrating and sleep deprivation (Figure 3). Some of the participants were recommended TMS therapy for their conditions by their physicians or friends who had experience engaging in psychoanalytic therapies before or through website advertisements. The participants stated that they were fed up of taking anti-depressant medications and they wanted to try something more helpful and effective to manage their conditions. As per the words of P10:

*“I was looking for different types of treatment because I felt vague, I had problems sleeping, I was no longer motivated, and had a lack of energy and I started looking on the Internet and I wanted to avoid drugs as much as possible because I heard that they give adverse effects or can give addiction”*

(P10).

It should be noted that all the participants accepted the treatment through TMS from a perspective of hope. Participants were willing to try something new with advanced technology other than the anti-depressant medications, which have side effects of their own. Each of the participants was willing to get out of that state of depression, sadness and detachment.

It was also observed that participants are interested in making this therapy more acceptable by integrating TMS as a standard treatment for psychiatric disorders. Participants believed that this therapy needed to be promoted more and that it should be an option in pharmacological treatment. There is also a need for better promotion so that people know about it in detail, and social media platforms can be used for promotion. One of the issues highlighted by participants was its insurance coverage, because it is a technologically new, advanced and relatively expensive procedure (P05).

*“I would promote it more and propose that it be reimbursed by the National Health Insurance Fund because many people believe they would benefit from this type of treatment. It is also a more modern treatment technique with such rapid results”*

(P05).

**Sub-Theme 2: Effectiveness**

Participants shared their views on whether they experienced challenges or benefits in TMS therapy. The participants had some concerns as it is a new technique, and did not know how they might react to the therapy (P19). Therapy helped most of the participants to reduce the symptoms of their condition, while for others it had a minimal effect and did not create changes in their mental state (P03).

*“At first I was cautious because I had never heard of this therapy and I was a little afraid of the possible effects, how my body might react to the therapy....But in addition to some tingling in the scalp or a mild, very mild migraine that was, anyway, transient immediately after the treatment, I can say that it had a positive impact on my mental state”*

(P19).

*“I did not experience very strong effects, but I felt the beginning of symptom relief, which helped me regain my emotional and inner balance. It was like a kind of trigger—one could call it ‘trigger therapy’”*

(P03).

**Figure 4:**
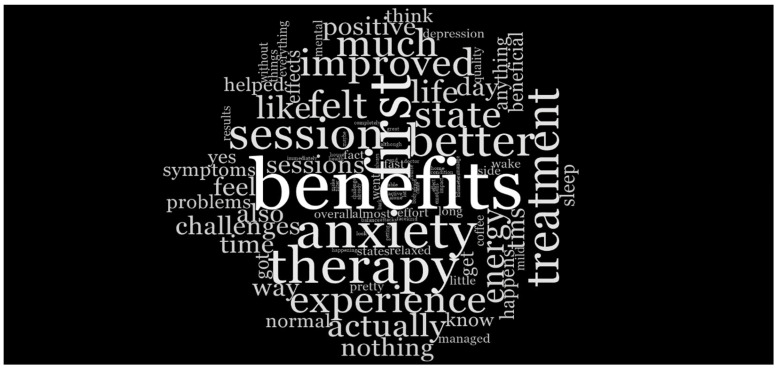
Word cloud for Sub-Theme 2, showing frequency of coded words.

The participants highlighted that TMS treatment influenced their daily lives and overall health in a very positive manner. The effective benefits of TMS treatments included an increased concentration level, problem-solving ability, awareness, emotional balance, panic control, declined anxiety, active energy, work–life balance, relaxation and overall improved quality of health (Figure 4).

P20 provided an insight into their daily life routine with decreased anxiety due to TMS in these words:

*“The decrease and disappearance of anxiety in a very large percentage, probably 90%, because everything that happens every day, if we look at them with an anxiety filter, happens badly”*

(P20).

*“Everything that happens in a day through the filter where this anxiety is missing happens completely differently, is much better, and, after all, the end of the day is satisfactory”*

(P20).

**Theme 2: Influential Factors in Deciding for TMS**

To answer the second research question, “What factors influence patients’ decision-making processes regarding the utilization of transcranial magnetic stimulation (TMS) for treating depression and AD with mixed anxiety and depressed mood in Romanian private practices? Participants were asked about their motivational factors within their social circle which helped them to choose TMS therapy. The social motivators in choosing TMS therapy included friends, colleagues, physicians’ recommendations and social networking platforms, as well as surfing the internet. Participants were willing to see the difference between the pharmacological treatment and any alternative treatment option. Participants were reluctant to take the medications because of their preconceptions of dependency, addiction and side effects. As stated by P04:

*“I was not willing to choose the pharmacological option. I would never have chosen pharmacological therapy to treat the symptoms. The fact that there are no side effects, that there is no risk of addiction”*

(P04).

TMS is a new, advanced alternative therapy which is an innovative treatment compared to classical or conventional therapies or treatments; hence, it should be given a chance to make an impact. The most important motivational factor in choosing TMS is its non-addictive characteristics. Most of the participants chose this therapy because they had the perception that TMS does not make them addicted to it. Some of the participants believed that conventional medication may have many side effects, creating addiction in long-term use (P05). On the other hand, they perceive new treatments like TMS as non-addictive, non-invasive treatments that can be successfully used in treatment of their condition, especially when pharmacological treatment does not work (P01). We can also observe that young people, especially, have a certain reservation regarding conventional treatments, and they focus on new and advanced therapies (P19).

*“I wanted to try something that could provide fast and reliable results, that, again, does not create dependency or cause other problems. And since I read about it on the internet… I decided to give it a try”*

(P05).

*“Being at a fairly young age, I did not want to start a conventional treatment, a medication that would require to be continued for many years. I wanted something that can be applied in the short term and that does not require years of therapy”*

(P19).

**The Role of Online Promotion**

**Figure 5:**
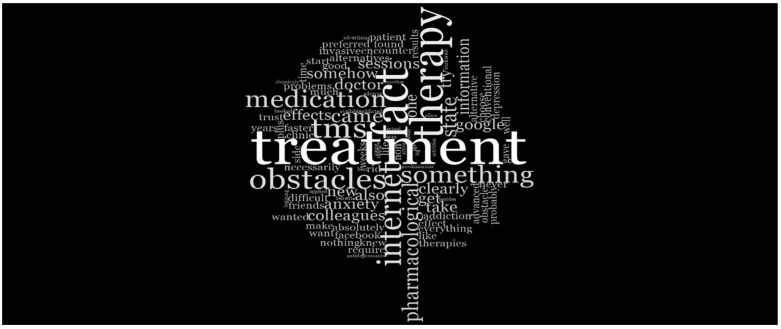
Word cloud for Theme 2, showing frequency of coded words.

Another considerable influential factor in patients’ decision for opting for TMS was the online promotion of this therapy on Google, social media and other sources from the internet. Even if recommended by friends, family or colleagues, patients ultimately used the internet to gather more information about this technique (Figure 5).

*“The previous treatment with medication did not work, and I found the TMS therapy clinic on Facebook”*

(P01).

*“I saw a lot of advertisements on Google and Facebook and decided that it would definitely be a good method”*

(P11).

*“Mainly my friend, and after that, the information I found available on Google”*

(P12).

*“Information—I searched on Google and the internet to find out what else I could do to recover”*

(P15).

*“My colleagues, plus the internet, because I also searched about it online”*

(P03).

**Obstacles in Accessing TMS Treatment**

While some participants had no difficulties accessing therapy, others faced challenges related to the clinic location and commuting.

*“No, I did not encounter any obstacles; everything went smoothly. I started being active again, having a generally good mood, and being efficient both in carrying out my activities and in everyday life”*

(P01).

However, a few participants reported that long distances and traffic congestion made it difficult to attend TMS sessions. Time management was another concern, especially for those with busy schedules.

*“As an obstacle, a significant one, though not necessarily major, was the fact that the location where I had to go for these therapies was quite far from me, and the traffic in Bucharest made it somewhat difficult for me to get to the therapy sessions. That was the only obstacle. Clearly, there were determining factors besides recommendations, and the fear of becoming dependent for life on conventional therapy somehow led me toward TMS”*

(P19).

*“Obstacles are about distance and the fact that there are 200 km in the middle and you can’t commute every time you do TMS. My schedule, which is quite crowded, doesn’t allow me to take a few weeks off to do this treatment, but if things were to evolve and if I reached the point when I would necessarily feel the need, I would do this and I would take the same decision to start treatment with antidepressants for anxiety. So clearly, I’d go to TMS variants”*

(P20).

**Theme 3: TMS Compared to Other Types of Treatments**

**Figure 6:**
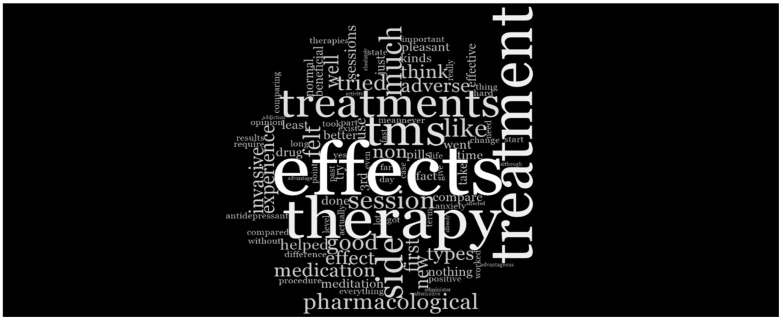
Word cloud Theme 3, showing frequency of coded words.

The participants provided their views and perceptions regarding the comparison of TMS and any other type of treatment. Many preferred TMS over conventional pharmacological treatments for their disorder. Some participants had previously tried other treatments, meaning that their opinions are not comparative. However, even in these cases, their perceptions of TMS remained positive (Figure 6).

It was observed that the perception of TMS was generally more favorable than that of traditional treatments. This preference was largely due to the belief that TMS has no side effects, does not cause addiction and is a safer alternative compared to drug therapy. One participant strongly advocated for TMS therapy over medication:

*“I did not try other types of treatments because I disagree with the pills, which is why I wanted to try this variant for anxiety, and finally it solved my problem without the need to take medication”*

(P05).

**Negative Perceptions of Pharmacological Treatments**

Most participants expressed strong reservations about pharmacological treatments due to concerns about addiction and side effects. Regardless of age, they were generally unwilling to opt for drug-based treatments. As one participant explained:

*“I have a very good opinion from the simple fact that this therapy has no side effects compared to pharmacological treatments and overall time of therapy is shorter than for medication”*

(P06).

Additionally, participants noted that TMS produced observable improvements more quickly than medication. While some individuals reported experiencing positive changes from the first sessions, others found the effects slower but still beneficial. However, some participants did not notice significant changes in their condition.

*“I did not do other types of treatments, but TMS is level 99, should be part of a program, at the national level, somewhat compensated and mandatory in certain situations, because of its efficacy. It’s very relevant to me personally”*

(P17).

*“I did not experience very strong effects, but I felt the beginning of symptom relief, which helped me regain my emotional and inner balance. It was like a kind of trigger—one could call it ‘trigger therapy’”*

(P03).

**Comparing the Speed and Side Effects of Medication vs. TMS**

A few of the patients were taking medication earlier along with the TMS therapy, and they stated that the difference was the time when TMS had a faster and quicker positive influence as compared to the medication, which is a longer and slower process. Some of the side effects of the medications were highlighted by the participants, but minimal side effects were mentioned concerning TMS. The side effects due to medications included drowsiness, nausea or other side effects that caused the patient to stop the pharmacological treatment.

*“Because of the medication, I had a state of drowsiness that affected my daily activities. With the help of rTMS, my health improved without any adverse effects”*

(P01).

*“In the past, I took pharmacological treatment and knew that it had side effects. At that time, it seemed much more advantageous to use this non-invasive procedure”*

(P07).

While some participants had personally experienced negative side effects from medication, others merely perceived medications as risky due to their potential for addiction and long-term dependency. However, regardless of whether participants had direct experience with pharmacological treatments, they did not associate TMS with any significant side effects.

*“I have a very positive opinion simply because this therapy has no side effects compared to pharmacological treatments, and the total duration of therapy is shorter than that of medication”*

(P06).

## 4. Discussion

This qualitative study examined the perceptions of patients with MDD and AD with mixed anxiety and depressed mood focusing on exploring effectiveness and accessibility, both influential factors in deciding for TMS, and how they view this therapy compared to other type of treatments. Overall, our findings indicate that TMS was effective in alleviating depressive symptoms and anxiety in both diagnostic groups, and was generally well-tolerated. A substantial proportion of patients achieved a clinically significant improvement or remission by the end of the TMS course [30], an outcome consistent with prior research on TMS in mood disorders. For example, a large multi-site observational study reported clinician-rated response and remission rates of approximately 58% and 37%, respectively, in pharmacotherapy-resistant depression [31]. Likewise, in our AD study [30], many patients experienced a marked symptom reduction, and in MDD study [29] the group that received pharmacological treatment and TMS obtained a 40% response rate, paralleling the ~30–60% response rates observed in real-world TMS practice. Such convergent findings reinforce that TMS can produce meaningful antidepressant effects, even in populations that have not responded to conventional treatments. Furthermore, we observed high treatment adherence with minimal dropouts, underscoring the acceptability of TMS. This is in line with controlled trials showing that dropout rates with TMS are low and comparable to those with sham treatment [32], suggesting that the procedure’s tolerability is high in clinical settings.

A key finding of our study is the high acceptability of TMS among participants, especially considering their reluctance to engage in pharmacological treatments. Many patients actively sought a non-medication alternative due to past experiences or concerns about antidepressant side effects. This mirrors observations in clinical practice where TMS is often pursued by individuals who cannot tolerate medication side effects or prefer to avoid long-term drug use. Participants in our studies expressed relief in having an effective option that did not carry sedation or other side effects commonly associated with antidepressants. Their positive attitudes towards TMS are consistent with reports of high patient satisfaction in TMS clinical trials, wherein dropout rates tend to be low, and patients often report a willingness to undergo the procedure again, if needed. Notably, initial skepticism about a “magnetic” treatment was overcome once patients experienced firsthand that TMS was painless and straightforward. This underscores that improving patient education about how TMS works can significantly increase its acceptability—a point highlighted in prior research where providing information boosted willingness to consider TMS as a treatment [33]. In our study, the combination of personal motivation to avoid drugs, information found online and clear counseling about TMS’s safety profile likely contributed to the strong acceptance of the therapy.

Patients’ decision-making in favor of TMS was often influenced by information encountered through social media and online channels. Several participants reported learning about TMS via internet research or social media. This trend reflects a broader phenomenon in which the popular media portrayal of TMS has been largely optimistic, framing it as a cutting-edge solution for hard-to-treat depression. This raises ethical concerns, because many marketing materials designed are too optimistic, and can create false expectations for the patients or make them pursue an expensive therapy that might not be suitable for them [34]. Such positive media coverage may have shaped patient expectations, instilling hope that TMS could succeed where medications had not. In the Romanian private healthcare context, where direct-to-consumer advertising is limited, many individuals turned to online health resources and social media to guide their treatment choices. The influence of these sources was double-edged: on one hand, success stories and news articles reduced stigma and fear around TMS, but on the other, overly enthusiastic portrayals sometimes led to unrealistically high expectations. This suggests that healthcare providers should be aware of the information patients are exposed to online and be prepared to discuss both the potential and limitations of TMS to align expectations with clinical reality.

Despite the clear benefits observed, the study also highlighted ongoing challenges and barriers to accessing TMS. Common barriers reported included the limited availability of TMS services locally, high out-of-pocket costs (in settings where insurance coverage was lacking), and the logistical burden of the treatment schedule. A standard TMS course requires daily (or near-daily) sessions over several weeks, which can be difficult for patients who live far from a TMS clinic or who cannot take time off work. Our findings echo the concerns raised in broader health system analyses: TMS remains virtually unavailable in many routine care settings, and even when offered, practical issues like funding for machines and staffing can cap the number of patients treated [35]. Patients in our study directly felt these constraints; several had to travel long distances or temporarily relocate to complete TMS, and a few, who were otherwise interested, could not pursue it earlier due to cost. Such barriers inevitably affect the real-world acceptability of TMS on a larger scale. Even though individual patients who receive TMS report high satisfaction and willingness to use it again, as observed in our sample, the treatment’s overall utilization remains low relative to the number of people who could potentially benefit. This gap between efficacy and uptake underlines an important point, demonstrating that TMS work is successful and necessary but not yet at a sufficient scale; issues of access and delivery must also be addressed. Our study, by incorporating patient feedback, suggests that overcoming these barriers (for example, through insurance coverage expansion, awareness campaigns or the development of more portable and time-efficient TMS protocols) could significantly increase the acceptability of TMS at the system level. Patients view TMS as a desirable alternative, so improving accessibility is crucial to translate TMS’s clinical efficacy into public health impact.

An important aspect of this research is the insight into patient perceptions of TMS as an alternative to pharmacotherapy. Many participants in our study explicitly cited dissatisfaction with antidepressant medications—whether due to inadequate relief or burdensome side effects—as a key motivation for pursuing TMS. These positive perceptions are in contradiction with the clinical results observed in our MDD study, where the group of patients that received only pharmacological treatment had an 80% clinical response rate [29]. This emphasis on seeking a non-pharmacological option echoes findings in the existing literature. A qualitative study involving 98 individuals who independently sought TMS treatment highlighted that the most frequently mentioned concern was the ineffectiveness of their current treatment. Additional key themes included actively searching for information regarding long-term illness management and a strong inclination to explore alternative therapies due to the adverse effects associated with prior treatments. Furthermore, many participants expressed an increasing sense of urgency to alleviate their symptoms, since their condition had continued to deteriorate despite conventional medical interventions [36]. Our study adds to this perspective by highlighting that when given the option, patients will opt for TMS not only for efficacy reasons, but also due to its favorable side-effect profile and the autonomy it offers them in managing their condition.

### Limitations

Several limitations of this study should be acknowledged when interpreting the results. First, the sample size was relatively small, which limits the power to detect subtle effects and may reduce the generalizability of the findings. The participants are only from Romania, which may not represent the diversity of experiences from a cultural point of view. The study design did not include a sham TMS or treatment-as-usual control group. Consequently, while patients showed significant improvement, we cannot definitively rule out placebo effects, spontaneous remission (especially in AD) or regression to the mean as contributing factors.

The lack of randomization and a control condition means that causality (that TMS per se caused the improvements) must be inferred cautiously. Additionally, our sample consisted of patients who were actively seeking TMS treatment, often after other therapies failed; this self-selection could bias the results toward more favorable perceptions of TMS. Participants knew they were receiving an active novel treatment, which may have elevated their expectations, and thus their reported outcomes (expectancy bias). Another consideration is the heterogeneity of the patient population, since we included both MDD and AD patients in the analysis. While this reflects a real-world clinical scenario where TMS might be tried in various psychiatric conditions, the pathophysiology and typical course of an acute stress-related AD differ from chronic or recurrent major depression. The pooling of these groups could have confounded the results, as AD patients might respond differently or even remit with time and supportive care alone. Our study was also limited to the acute treatment outcomes; we did not systematically follow patients long-term to assess the durability of TMS benefits or the need for maintenance treatments. Finally, future research studies could benefit from incorporating quantitative data and provide a more balanced view of TMS efficacy and acceptability.

## 5. Conclusions

In conclusion, our study demonstrates that rTMS is an effective and generally acceptable treatment option for patients suffering from MDD, as well as for those experiencing AD with mixed anxiety and depressed mood. Patients treated with TMS showed significant improvements in depressive and anxiety symptoms, and importantly, they regarded TMS as a favorable alternative to traditional pharmacotherapy. Many participants were drawn to TMS due to dissatisfaction with antidepressant medications or their side effects, and their positive clinical outcomes reinforce the notion that TMS can fill a critical therapeutic gap for such individuals. These patient-reported experiences, when compared with the broader literature, consistently indicate that TMS offers substantial antidepressant benefits without compromising tolerability [32]. Our findings thus align with accumulating evidence from clinical trials and meta-analyses demonstrating that TMS is a safe, efficacious and patient-friendly intervention for specific psychiatric disorders.

Based on both our results and the existing research, TMS has a valuable role to play in clinical practice, particularly for patients who have not responded to medications or who prefer non-pharmacologic treatments. Wider implementation of TMS could enhance treatment outcomes in difficult-to-treat depression and provide relief for patients who otherwise have limited options. However, the real-world deployment of TMS will require continued efforts to improve accessibility, including increasing the availability of TMS centers, securing insurance coverage and educating both clinicians and patients about this therapy. Future studies should further explore the efficacy of TMS in diverse populations and seek to optimize treatment protocols and delivery methods. Such research will help to confirm and extend TMS’s therapeutic potential and ensure that its benefits are accessible to the broadest possible range of patients. Ultimately, our study supports the growing consensus that TMS is a promising and viable antidepressant treatment modality, and with ongoing investigation and improved access, it could become an integral component of standard care for depression and related disorders.

## Figures and Tables

**Table 1:** Demographic information and coding of study participants.

Code	Sex	Age in years
P01	F	21
P02	F	51
P03	F	39
P04	F	30
P05	F	28
P06	F	28
P07	F	35
P08	F	41
P09	F	40
P10	F	28
P11	F	29
P12	F	44
P13	F	61
P14	F	64
P15	F	26
P16	F	31
P17	M	36
P18	M	35
P19	F	25
P20	F	42

**Table 2:** Age distribution of the study participants.

Descriptive Statistic		Major Depressive Disorder	AD with Mixed Anxiety and Depressed Mood
Age			
N	Valid Data	10	10
	Missing Data	0	0
Mean		42.90	33.00
Minimum		21	25
Maximum		64	44

## Data Availability

Privacy restrictions are applied for 2 years according to university request.
